# Bilateral cellulitis caused by invasive aspergillosis associated with bilateral intraorbital abscesses: a case report

**DOI:** 10.1186/s12886-020-01606-7

**Published:** 2020-08-15

**Authors:** Jiahui Wu, Hao Zhou, Ruili Wei, Jinwei Cheng

**Affiliations:** 1 Department of Ophthalmology, Shanghai General Hospital, Shanghai Jiao Tong University School of Medicine, Shanghai, China; 2 Shanghai Engineering Center for Precise Diagnosis and Treatment of Eye Diseases, Shanghai Engineering Center for Visual Science and Photomedicine, National Clinical Research Center for Eye Diseases, Shanghai Key Laboratory of Ocular Fundus Diseases, Shanghai, China; 3 Department of Ophthalmology, Shanghai Changzheng Hospital, Second Military Medical University, Shanghai, China

**Keywords:** Intraorbital abscess, Orbital cellulitis, Aspergillosis, Fungal infections

## Abstract

**Background:**

Orbital invasive aspergillosis infection is rare life-threatening infection, most commonly seen in immunocompromised patients and extremely rare in individuals without risk factors. Here we present a rare case of bilateral cellulitis caused by invasive aspergillosis associated with bilateral intraorbital abscesses in a female patient.

**Case presentation:**

A 49-year-old woman presented with a 3-month history of painful proptosis and periorbital swelling of bilateral eyes. She was initially diagnosed as bilateral orbital cellulitis complicated with cavernous sinus thrombosis and was treated with antibiotic medication for 1 month, but her symptoms persisted. MRI demonstrated orbital masses behind both globes. The lesion in right orbit was biopsied with a diagnosis of orbital granulomatosis with invasive aspergillosis infection. The patient was healed after receiving antifungal treatment.

**Conclusions:**

This is an unusual case about bilateral orbital abscesses with invasive fungal infection. Fungal infection of the orbit should be considered when patient does not respond to combination of anti-inflammatory and antibiotic therapies, even in some cases without any risk factors.

## Background

Orbital fungal infections are rare and often life-threatening, with mortality ranging from 21 to 80% [1]. Because of the resemblance of acute orbital bacterial infections and lack of clinical manifestations, invasive fungal infection is difficult to be diagnosed, which usually cause delay and improper treatment, such as corticosteroids therapy [2].

To date, there are only a few published reports about individuals having bilateral orbital involvement due to extension of fungal sinusitis [3, 4]. To our knowledge, there is even limited published case about bilateral intraorbital abscesses caused by invasive fungal infections in patients without any risk factors, such as immunocompromising conditions. Here we present an unusual case of bilateral cellulitis caused by invasive aspergillosis associated with bilateral intraorbital abscesses in an adult.

## Case presentation

**Fig. 1:**
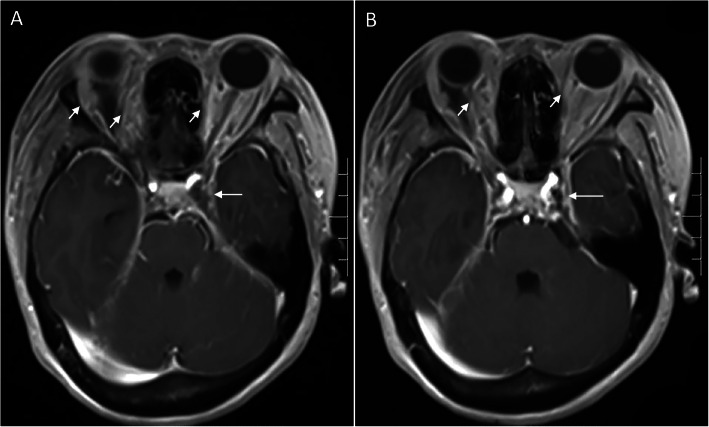
T1 contrast-enhanced MRI showed bilateral orbital lesions (short arrows) spreading to the cavernous sinus (long arrows)

A 49-year-old woman presented with a 3-month history of painful proptosis and periorbital swelling of bilateral eyes, accompanying with reduced vision and diplopia. The patient declared no medical history, such as hypertension, diabetes and systemic immunosuppression. She also denied recent trauma. She had been treated with antiviral medication and corticosteroids for 5 days in another hospital as the initial diagnosis was herpes virus infection, but her symptoms deteriorated. The vision at first visit was 20/25 OD and 20/30 OS. The axial and sagittal T1 contrast-enhanced magnetic resonance imaging (MRI) showed bilateral orbital lesions spreading to the cavernous sinus (Fig. 1). She was therefore diagnosed as bilateral orbital cellulitis complicated with cavernous sinus thrombosis. Thereafter, she was given antibiotic therapy for 1 month and her symptoms had been improved but not healed.

**Fig. 2:**
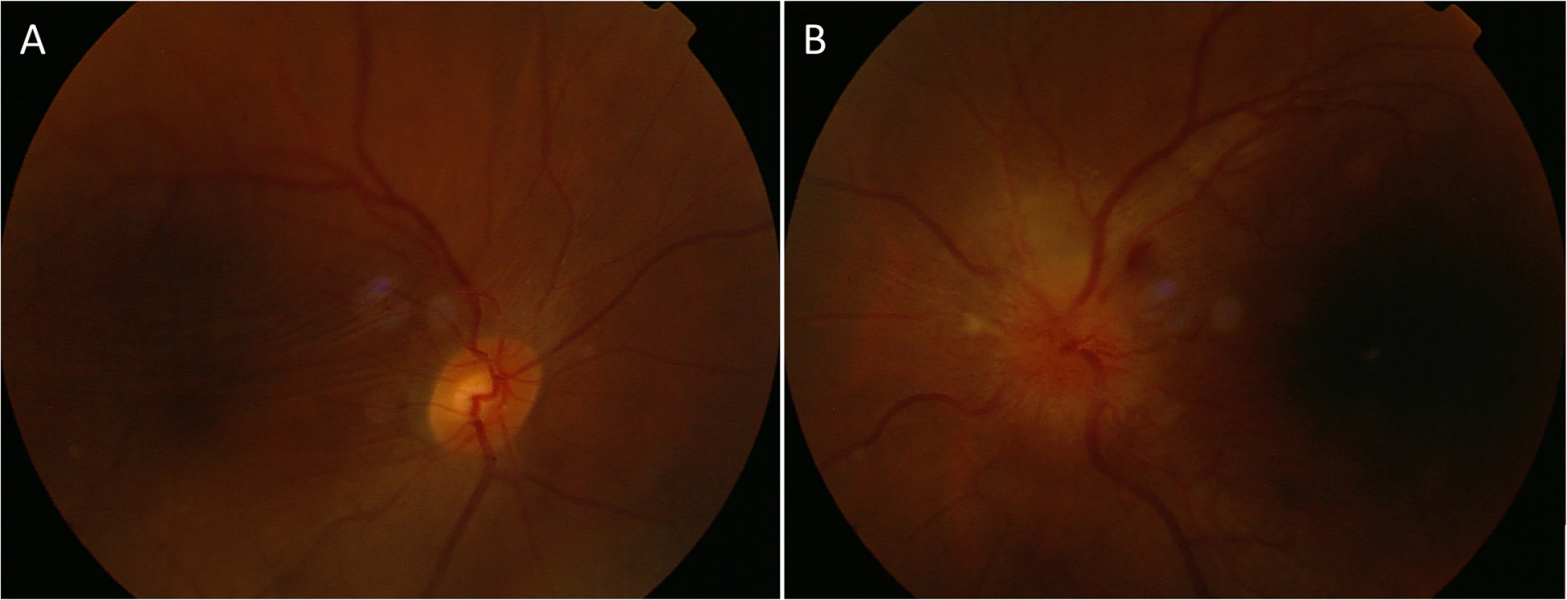
Respectively represents fundus photography of both eyes. **a** Choroidal folds in the right eye. **b** Optic disc swelling with lamellar hemorrhage around optic disc in the left eye

**Fig. 3:**
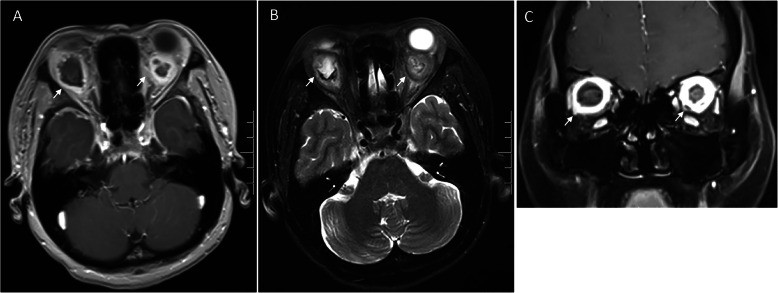
**a** T1-weighted contrast-enhanced imaging showed a predominantly low intensity signal with mildly heterogeneous in center of the masses behind both globes (short arrows). **b** T2-weighted image demonstrated a heterogeneous hyper intense signal behind each globe (short arrows). **c** Coronal plane image illustrates hyperintense signal around both globes (short arrows)

On follow-up examination, best-corrected visual acuities remained as 20/25 OD and 20/30 OS. Intraocular pressures were 21 mmHg OD and 19.3 mmHg OS. Ophthalmologic examination revealed bilateral exophthalmos, bulbar conjunctival edema and conjunctival congestion. Ocular alignment showed a limitation on elevation and abduction of both eyes. Corneas were clear with quiet anterior chamber. Both pupils were round, but there was an afferent pupillary defect in the left eye. Choroidal folds observed in the right eye, and optic disc swelling with lamellar hemorrhage around optic disc was found in the left eye (Fig. 2). MRI demonstrated orbital masses behind both globes (Fig. 3). T1-weighted contrast-enhanced imaging showed a predominantly low intensity signal with mildly heterogeneous in center of the masses. T2-weighted image demonstrated a heterogeneous hyperintense signal behind each globe. The patient received a number of blood tests, but no significant abnormality was detected. The C-reactive protein level and erythrocyte sedimentation rate were in normal range. The immunity tests showed negative in antistreptolysin-O, and rheumatoid factors (RF), including IgM-RF, IgA-RF, IgG-RF. The routine blood test also showed nothing abnormal, the results of white blood cell count, red blood cell count, haemoglobin, lymphocyte count, neutrophil count and platelet were all in normal range. The only finding was that eosinophil count was 0.72 × 10^9/L which was slightly above normal range.

Lateral orbitotomy was next performed on the patient’s right eye. A hard mass sized 1.5 × 1.5 × 2 cm was excised with accompanying amounts of pus liquid and black necrotic tissue. Histopathological examinations diagnosed orbital granulomatosis with invasive aspergillosis infection. The patient eventually received 3-week anti-fungal therapy, itraconazole 200 mg twice a day by oral administration. Her symptoms gradually resolved.

**Fig. 4:**
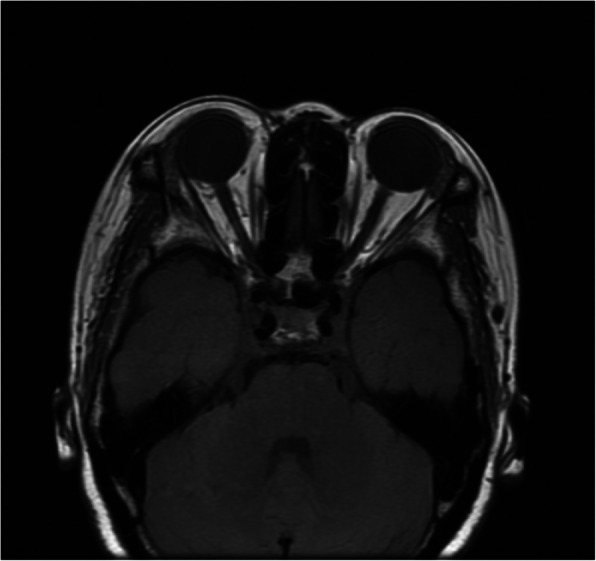
T1-weighted MRI image taken at 3-year follow-up shows no sign of orbital lesions behind both globes

At the 8-month follow-up, the patient’s vision got improved to 20/20 OD and 20/20 OS, findings from ophthalmic examination were unremarkable. MRI scan demonstrated that both orbital lesions regressed (Fig. 4). She so far remained clinical stable for 3 years.

## Discussion and conclusions

Orbital lesion presented as a granulomatous manifestation is usually caused by orbital inflammation. Orbital granulomatosis can be a solely disease or part of a systemic disease, such as antineutrophil cytoplasmic antibody (ANCA)-associated vasculitides [5]. However, orbital infection also sometimes presents as granuloma formation, which can be caused by various infections, such as tuberculosis, syphilis, parasites, and fungus. Therefore, incisional biopsy with pathological examination is sometimes necessary for diagnosis when clinical and radiological findings of an orbital inflammatory mass are inconclusive [6]. While in this case, the patient had symptoms improved after antibiotic therapy, but her symptoms were not completely controlled and MRI scan demonstrated that bilateral orbital lesions did not decrease, which suggested there were mixed infections and other diagnosis rather than orbital inflammation. Therefore, we performed lateral orbitotomy for further investigation. However, we only did histopathological examination but without microbiological evaluation which was a limitation. This case is a good example that microbiological evaluation is necessary whenever there is suspicion of infection.

Invasive fungal infections of the orbit are rare but often fatal, and patients with mucormycosis had a higher mortality than patients with aspergillus [1]. Invasive aspergillosis is most commonly seen in immunocompromised patients with the primary risk factors, such as neutrophil defects and long-term corticosteroid treatment [7]. Although rarely, aspergillosis also might be seen in individuals without risk factors. There were two reported cases about fungal sinusitis associated with bilateral orbital involvement [3, 4]. While this presented case is about bilateral orbital cellulitis caused by invasive aspergillosis associated with bilateral intraorbital abscesses in a patient without any risk factors. However, we suspected the original cause could be fungal ethmoiditis, because her MRI images showed a hyper intense signal at ethmoid sinus (Fig. 1). It is possible that the fungal ethmoiditis further spread to orbit and then developed to intraorbital abscesses.

Orbital imaging of fungal infection commonly demonstrated an ill-defined mass or infiltration of orbit [8]. MRI provides excellent contrast resolution images of the orbit. The enhanced MRI is essential for distinguishing orbital infections, especially orbital abscess, from edema and noninfectious inflammatory mass lesions. It is widely accepted that T1 weighted image with gadolinium-based contrast enhancement and fat saturation is the “gold standard” for detecting orbital abscess [9].

The lack of standard guideline of orbital fungal infection makes it difficult to be handled in clinic, antifungal therapy alone cannot be enough to control some severe cases, so surgery associated with antifungal therapy should be considered in this situation. Prompt treatment is also essential as fungal infection is invasive, which could cause irreversible damage. Management often begins with surgical debridement followed by systemic antifungal drug therapy [2, 7].

In conclusion, fungal infection of the orbit should be considered when patient does not respond well to the combination of anti-inflammatory and antibiotic therapies. Orbital MRI is essential for diagnosis, if the images demonstrate unusual findings, then biopsy is necessary.

## Data Availability

All data generated or analyzed during this study are included in this published article.

## Abbreviations

MRI: Magnetic resonance imaging
ANCA: Antineutrophil cytoplasmic antibody
